# Optimized biosynthesis of lytic enzymes by special *Trichoderma citrinoviride*

**DOI:** 10.1007/s11356-024-35251-0

**Published:** 2024-10-12

**Authors:** Michał Piegza, Wojciech Łaba

**Affiliations:** Department of Biotechnology and Food Microbiology, Wroclaw University of Environmental and Life Sciences, Wrocław, Poland

**Keywords:** Biocontrol, Lytic enzymes, Box-Behnken design

## Abstract

The use of *Trichoderma* filamentous fungi in the wide concept of biocontrol is still a highly relevant topic. The multifaceted nature of their impact on phytopathogenic microorganisms results from the species diversity and complexity of their antagonistic action. The presented research aimed to determine optimal cultivation conditions of two *T. citrinoviride* strains for the biosynthesis of major enzymes especially those involved in the biocontrol process. Culture conditions were optimized using a three-factor Box-Behnken design to maximize the yield of chitinase and lichenase. The following independent variables were included in the model: incubation temperature, initial pH, and supplementation with fungal biomass. As a result of statistical optimization, unprecedented activities of extracellular lytic enzyme were achieved. For the B1 and B3 strains, the optimal pH was 3.5 or 7.5, respectively, in the determination of chitinase biosynthesis. It was similar for the biosynthesis of β-1.3 and β-1.4 glucanases, but at higher cultivation temperature. The exception was the B3 strain, for which the optimal pH in glucanase biosynthesis was 5.5. The most stimulating culture temperature in the process of chitinase biosynthesis and β-1.3 and β-1.4 glucanases was above 25 °C. In that, the levels of enzyme biosynthesis and corresponding composition culture environment were confirmed to be strain-dependent.

## Introduction

Fungi of the genus *Trichoderma* are commonly found in the environment in all types of soil. They colonize the root system of various crops and wild plants (Kancelista et al. 2013). Although they dominate in a variety of soils, including agricultural, forest, and desert soils in all climatic zones, they exhibit some kind of territorial specificity. Within this genus, more than 260 species are currently listed, but about only 35 species are of biotechnological importance due to their ability to produce lytic enzymes (exoglucanase, endoglucanase, chitinase, and protease) and antibiotics and a number of volatile compounds, including alcohols, aldehydes and ketones, ethylene, hydrogen cyanide, and monoterpenes, as well as non-volatile compounds, including gliotoxin (Sharma et al. 2019; Mambaeva et al. 2019). *Trichoderma* sp. is used as a biological control measure against soil-borne diseases that cause economic losses in crops mainly due to the ability to degrade the cell wall of phytopathogens (Al-Shuaibi et al. 2024, El-Sobky et al. 2019, Ribero et al. 2019, Vargas-Inciarte et al. 2019, Zhao D et al. 2023). The paramount factor for the production of enzymes by fungi is the type of carbon source available. The production of enzymes is also influenced by lighting conditions, growth and secretion rates, and stress. *Trichoderma* isolates produce an array of hydrolytic enzymes i.a. chitinases, β-1,3- and β-1,6-glucanases, and proteases to facilitate chitin or total fungal cell walls as a source of carbon. *T. hamatum* strains display high antimicrobial activity due to the presence of specific β-glucanase and chitinase, which play a major role during cell wall degradation (Sharma et al. 2019). Microbial chitinases, especially of fungal origin, have found applications in the recycling of chitin-rich shell and shellfish waste. Chitinase expression is induced naturally in order to achieve maximum enzyme production; however, proper culture conditions must be maintained. Among factors affecting the production of this extracellular enzyme are carbon and nitrogen sources, salts, aeration, medium pH, fermentation, temperature, and humidity. Optimization of the given factors was carried out either using a classic one variable at a time (OVAT) or a statistical experimental design approach (Halder et al. 2019; Dukariya and Kumar 2021). Exogenous β-1,3–1,4-glucanases are used to reduce the viscosity of barley complexes of β-glucans during the mashing process in the brewing industry and can also improve the digestibility of β-glucans in poultry and swine feed. The cell walls of sugar cane are rich in β-glucans, and the presence of β-1,3–1,4-glucanases in mixtures with saccharifying enzymes aids the release fermentable sugars from lignocellulose, they are used as a raw material for the production of biofuels (Furtado et al. 2011).

The aim of the study was to model and optimize the biosynthesis of lytic enzymes classified as chitinases and beta-1,3(1,4)-glucanases by very selected strains of *Trichoderma citrinoviride* employing response surface methodology.

## Data and methodology

### Microbial strains and culture conditions

Two strains of *Trichoderma citrinoviride* used in the study, designated as C1 and B3, originated from the Culture Collection of the Department of Biotechnology and Food Microbiology of the Wrocław University of Environmental and Life Sciences, Wrocław, Poland (Witkowska et al. 2009; Piegza et al. 2021).

The base culture medium was composed of fodder yeast 1 g/L, sugar beep pulp 1 g/L, and malt extract 0.4 g/L. In order to establish the optimal conditions for the biosynthesis of lytic enzymes, the medium was prepared in three variants of fungal biomass content: 0.2 g/20 mL; 0.6 g/20 mL; 1.0 g/20 mL. The additional surveyed factors were pH, with values of 3.5, 5.5, and 7.5, and temperature, with values of 15 °C, 25 °C, and 35 °C. The medium was dispensed in the amount of 20 mL in 250 mL conical flasks.

The cultures were carried out in agitated conditions at 160 rpm.

### Analytical determinations

The activity of β-1,3-glucanases and chitinases was determined with 0.5% laminarin and 4% chitin as substrates, respectively, in 0.05 M acetic buffer pH = 4.8 by a method involving DNS reagent (Piegza et al. 2014). Enzyme activities (chitinases, glucanases) were expressed in nKat/mL. Results were presented as average values of duplicates.

### Box-Behnken experimental design

The process of glucanase and chitinase production by *Trichoderma citrinoviride* B3 and C1 was modeled separately using a 3-variable Box-Behnken design. The design based on multiple regression employing a quadratic polynomial is meant to describe causal relationships between independent variables, including their interactions, and the response. The following polynomial equation (Eq. 1) was built for each model:

1 $$Y = {\beta }_{0}+ {\beta }_{1}{X}_{1} + {\beta }_{2}{X}_{2} +{\beta }_{3}{X}_{3}+{\beta }_{11}{X}_{1}{X}_{1}+{\beta }_{22}{X}_{2}{X}_{2}+{\beta }_{33}{X}_{3}{X}_{3}+{\beta }_{12}{X}_{1}{X}_{2}+ {\beta }_{13}{X}_{1}{X}_{3}+ {\beta }_{23}{X}_{2}{X}_{3}$$

where *Y* — the predicted response; β_0_ — the intercept; β_1_, β _2_, β_3_ — linear regression coefficients; β_11_, β_22_, β_33_ — quadratic regression coefficients; β_12_, β _13_, β_23_ — interaction terms.

The experimental design layout comprised the main and interaction effects of culture medium pH (X_1_), cultivation temperature (X_2_), and supplementation with fungal biomass (X_3_), in relation to the measured response (enzymatic activity) (Tables 1, 2). The actual values of independent variables are listed in Table 3.

**Table 1:** Experimental layout of the Box-Behnken design, with the obtained response outcomes, transformed responses, and predicted responses for the *T. citrinoviride* C1

Run	Independent variables			Glucanase activity [nKat]			Chitinase activity [nKat]		
	Temperature [°C]	pH	Biomass [g]	Actual	Transformed	Predicted	Actual	Transformed	Predicted
1	− 1	− 1	0	0.229	0.0896	0.2664	0.203	0.080	− 0.001
1	− 1	0	+ 1	2.003	0.4776	0.3605	0.180	0.072	0.161
3	− 1	0	− 1	0.112	0.0460	− 0.1273	0.271	0.104	0.198
4	− 1	+ 1	0	0.399	0.1458	0.2593	0.969	0.294	0.168
5	0	− 1	− 1	3.009	0.6030	0.8136	0.385	0.141	0.272
6	0	− 1	+ 1	14.970	1.2032	1.3014	0.110	0.045	0.259
10	0	− 1	+ 1	6.537	0.8772	0.8018	0.401	0.146	0.234
11	0	+ 1	+ 1	24.466	1.4060	1.2896	0.340	0.127	0.222
12	+ 1	− 1	0	16.142	1.2341	0.9551	3.526	0.656	0.519
13	+ 1	0	− 1	2.300	0.5185	0.5566	1.020	0.305	0.537
14	+ 1	0	+ 1	7.113	0.9092	1.0445	1.731	0.436	0.499
15	+ 1	+ 1	0	5.808	0.8330	0.9386	2.217	0.508	0.325
7	0	0	0	18.024	1.2793	1.1979	2.319	0.521	0.355
8	0	0	0	16.300	1.2380	1.1979	2.034	0.482	0.355
9	0	0	0	14.603	1.1932	1.1979	2.471	0.540	0.355

**Table 2:** Experimental layout of the Box-Behnken design, with the obtained response outcomes, transformed responses, and predicted responses for the *T. citrinoviride* B3

Run	Independent variables			Glucanase activity			Chitinase activity	
	Temperature [°C]	pH	Biomass [g]	Actual [nKat]	Transformed	Predicted	Actual [nKat]	Predicted
1	− 1	− 1	0	0.008	0.0032	0.0722	0.146	0.199
2	− 1	0	+ 1	2.129	0.4954	0.6323	0.209	0.204
3	− 1	0	− 1	3.085	0.6112	0.4607	0	0.060
4	− 1	+ 1	0	0.419	0.1519	0.0966	0.281	0.174
5	0	− 1	− 1	3.127	0.6157	0.6077	0.534	0.401
6	0	− 1	+ 1	6.864	0.8957	0.7793	0.203	0.376
7	0	+ 1	− 1	5.905	0.8392	0.9966	0.503	0.545
8	0	+ 1	+ 1	14.896	1.2013	1.1682	0.601	0.520
9	+ 1	− 1	0	0.798	0.2549	0.3102	0	0.040
10	+ 1	0	− 1	10.540	1.0622	1.0632	0	− 0.099
11	+ 1	0	+ 1	15.675	1.2221	1.2347	0	0.045
12	+ 1	+ 1	0	12.568	1.1325	1.0636	0	0.015
13	0	0	0	11.602	1.1004	1.1467	0.950	0.987
14	0	0	0	14.519	1.1909	1.1467	0.910	0.987
15	0	0	0	13.085	1.1488	1.1467	1.100	0.987

**Table 3:** Experimental values of independent variables

	− 1	0	+ 1
X_1_: temperature [°C]	15	25	35
X_2_: pH	3.5	5.5	7.5
X_3_: fungal biomass [g]	0.2	0.6	1.0

Prior to the assessment of resultant models with analysis of variance (ANOVA), the responses were evaluated using the Box-Cox statistics to verify the need for data transformation (Vélez et al. 2015). Where applicable, the responses were subjected to decimal logarithm transformation after increment of response values by 1. Furthermore, the obtained regression models were reduced to insignificant terms (*p* > 0.05). Insignificant linear terms were not reduced in the case when their quadratic or interactive components were significant. The experimental design, polynomial equation fit, regression, and ANOVA statistics, as well as the response optimization analysis were performed with Statistica 14 software (TIBCO Software Inc., USA).

## Results

**Fig. 1:**
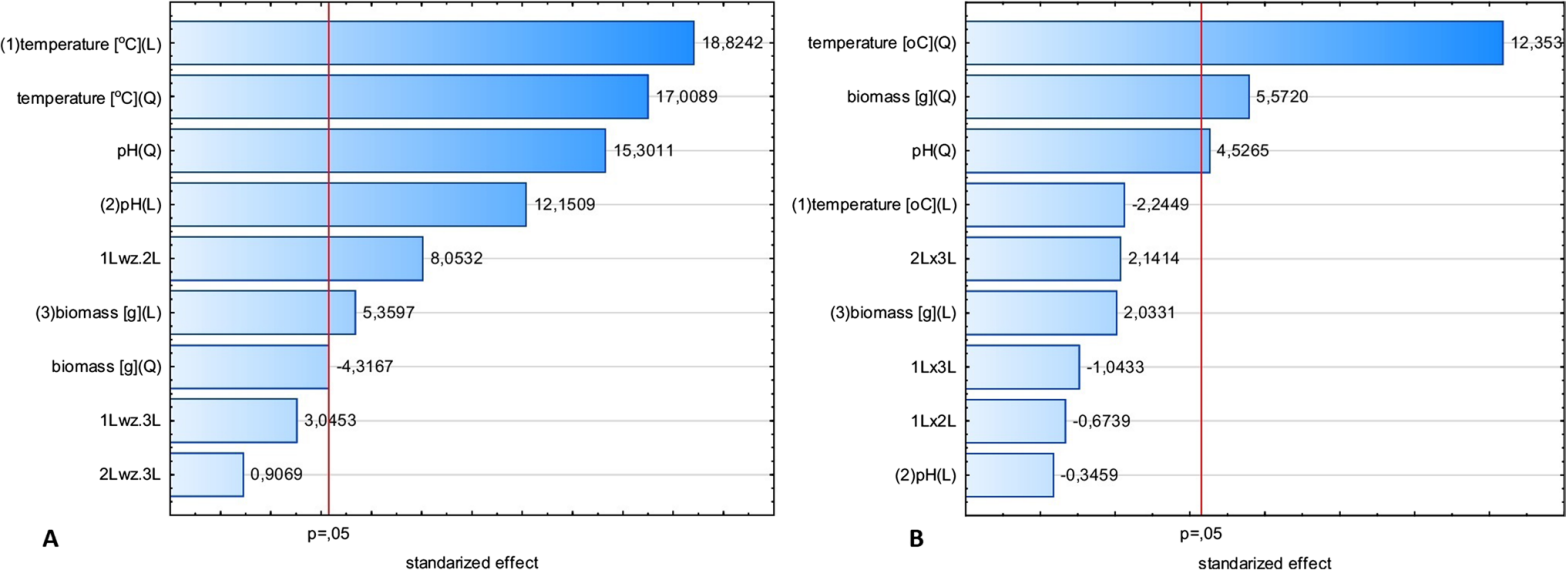
Pareto graphs of standardized effects of the Box-Behnken design for the *T. citrinoviride* B3 strain, prior to the reduction of models: **a** glucanase, after data transformation, (**b**) chitinase

**Fig. 2:**
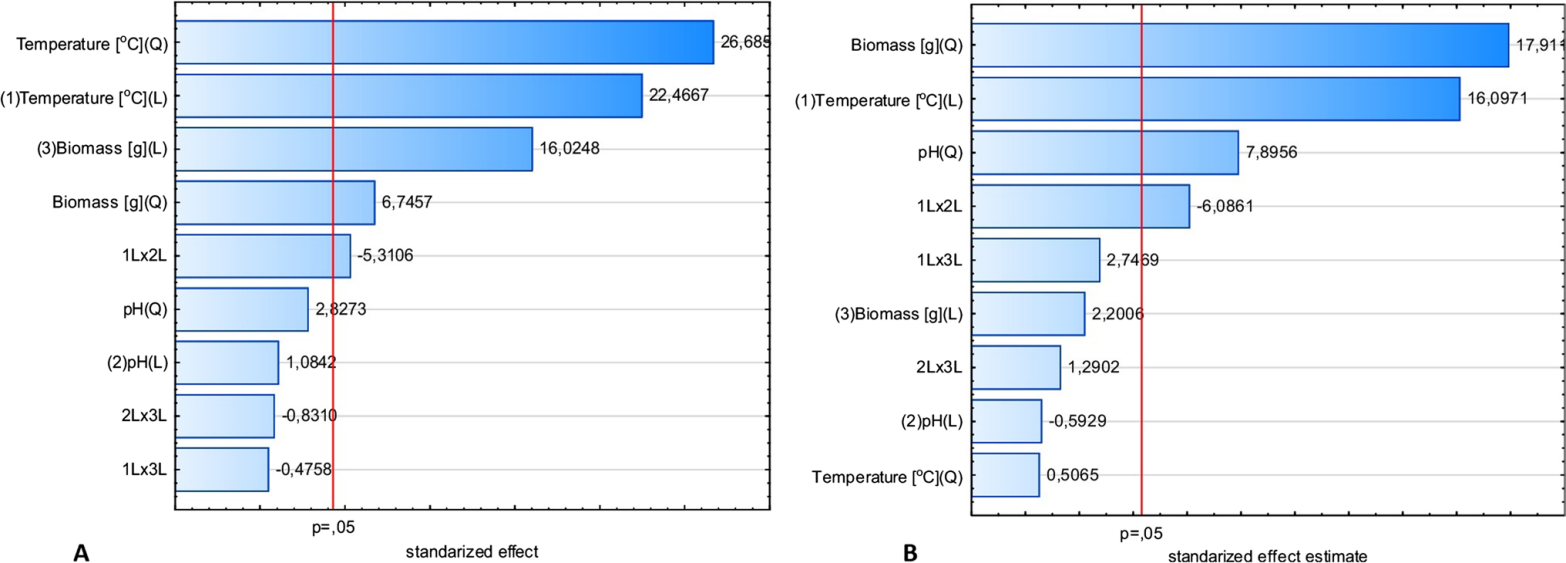
Pareto graphs of standardized effects of the Box-Behnken design for the *T. citrinoviride* C1 strain, after data transformation, prior to the reduction of models: **a** glucanase, **b** chitinase

A three-variable experimental design according to Box-Behnken was employed to determine the empirical relationships between culture parameters, i.e., temperature, pH of medium, and supplementation with fungal biomass, to exert impact on the production of extracellular glucanase and chitinase by two strains of *Trichoderma citrinoviride* (Figs. 1, 2). The Box-Cox transformation statistics for the models (Table 4), except that of chitinase of the B3 strain, confirmed that, implying from the significant Chi^2^ value, the residual sum of squares could be reduced due to the transformation of the response. Hence, the responses were subjected to a transformation, prior to the evaluation with analysis of variance (ANOVA).

**Table 4:** Box-Cox transformation statistics of responses

Strain/enzyme	Response	λ	MSE	SSE	Chi^2^	*p*
B3 glucanase	Actual response	0.5067	2.1286	10.3723	11.1070	0.0009
	Transformed response	1.0625	0.0020	0.0794	0.1275	0.7210
B3 chitinase	n.a.*	-	-	-	-	-
	-	-	-	-	-	-
C1 glucanase	Actual response	0.0212	2.9249	22.3261	29.9890	0.0000
	Transformed response	0.6379	0.0019	0.2322	2.7056	0.1000
C1 chitinase	Actual response	0.4490	0.0492	0.2258	18.3211	0.0000
	Transformed response	0.858 9	0.0009	0.0114	0.9263	0.3358

*Box-Cox statistics are unavailable due to the occurrence of null values of the response

**Fig. 3:**
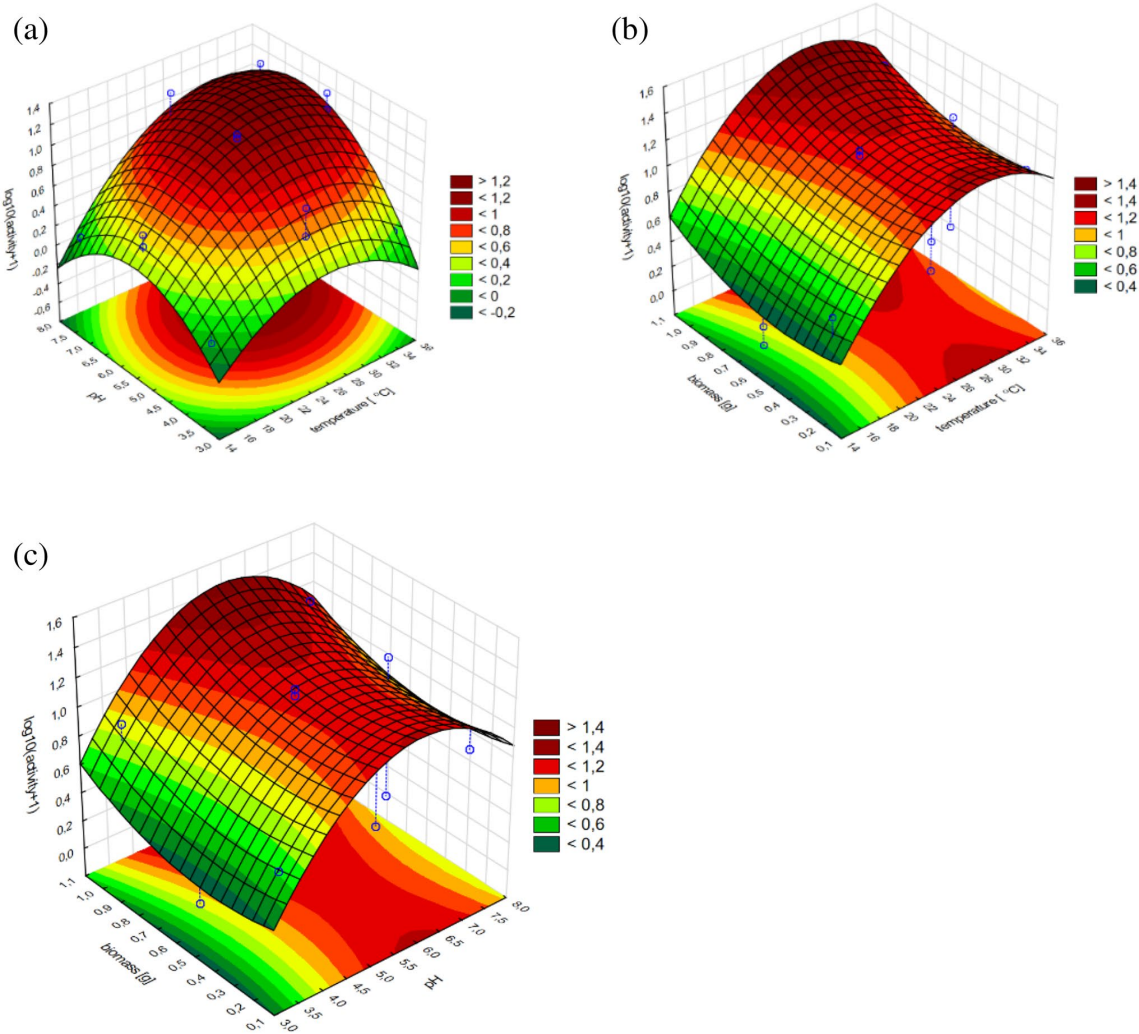
Response surface plots fitted to the model describing glucanase production by *T. citrinoviride* B3, with regard to combined effects of (**a**) culture medium pH and cultivation temperature; **b** addition of fungal biomass and cultivation temperature; **c** addition of fungal biomass and pH of the medium

Introductory analysis of standardized effects from the obtained regression models concerning *T. citrinoviride* B3 allowed us to confirm that virtually each of the evaluated independent variables significantly affected both, glucanase and chitinase activity. The highest impact on the glucanase production was exerted by culture temperature and pH of the culture environment, including both, linear and quadratic effects. Lower effects were bound to the content of the fungal biomass supplement, of mainly linear characteristics. Moreover, a significant interaction was found between pH and culture temperature. The response surface plots allow to assess variation of the dependent variable in function of selected independent variables. In the case of glucanases of the B3 strain, the combined effect of culture medium pH and cultivation temperature produced an unambiguous peak, implying that optimum values for these parameters were determined (Fig. 3). Also, the proportional, but not linear, impact of the fungal biomass addition was found. The highest glucanase production occurred at the maximum evaluated addition of the biomass.

As to the production of chitinase by the B3 strain, mostly culture temperature and fungal biomass were strongly influential; however, a minimal, but significant impact was denoted for the pH of the culture medium. All three parameters exhibited quadratic characteristics, which allowed us to determine their optimal values (Table 5).

**Table 5:** Optimal levels of input variables inferred from each model

	*T. citrinoviride* B3		*T. citrinoviride* C1	
	Glucanase	Chitinase	Glucanase	Chitinase
X_1_: temperature [°C]	28	24	28	35**
X_2_: pH	6.7	5.4	5.5*	4.6
X_3_: biomass [g]	0.8	0.6	0.9	0.2**
Predicted maximum activity [nKat]	19.91	9.86	22.67	3.63

*Insignificant influence over the experimental range

**The value of experimental boundary

**Fig. 4:**
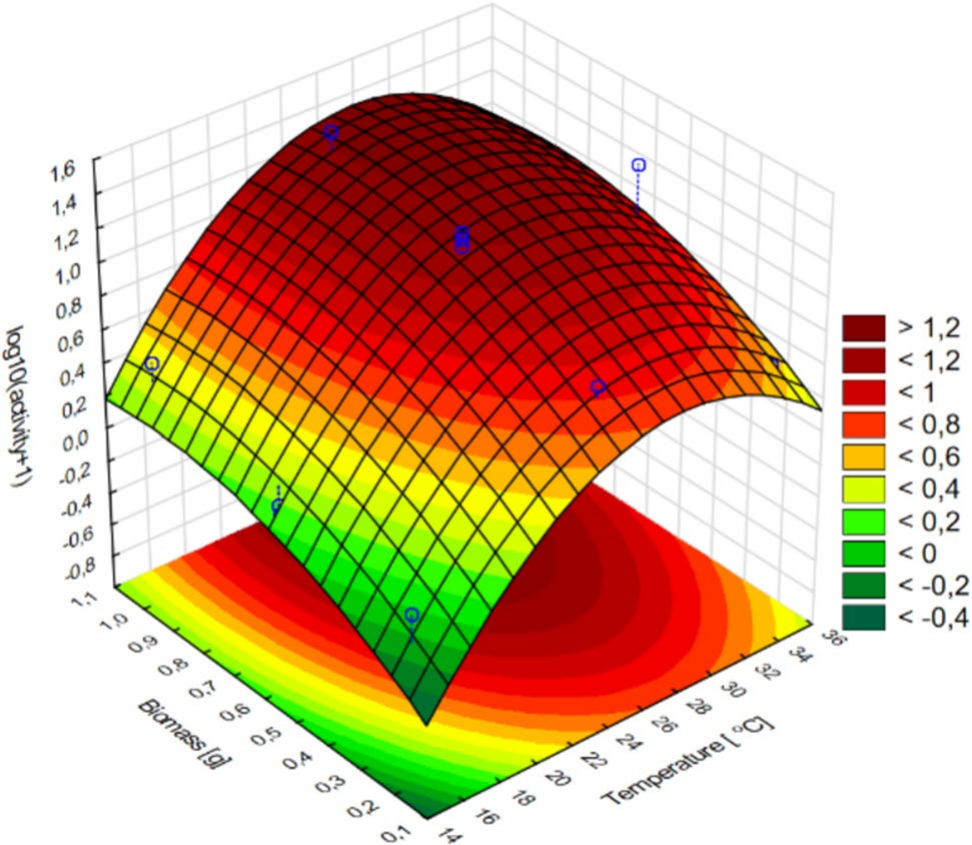
Response surface plots fitted to the model describing glucanase production by *T. citrinoviride* C1 with regard to the addition of fungal biomass and cultivation temperature

On the contrary, the production of glucanase by *T. citrinoviride* C1 appeared to be dependent on culture temperature and biomass (linear and quadratic terms); however, no statistically significant impact of pH was confirmed (Fig. 4).

**Fig. 5:**
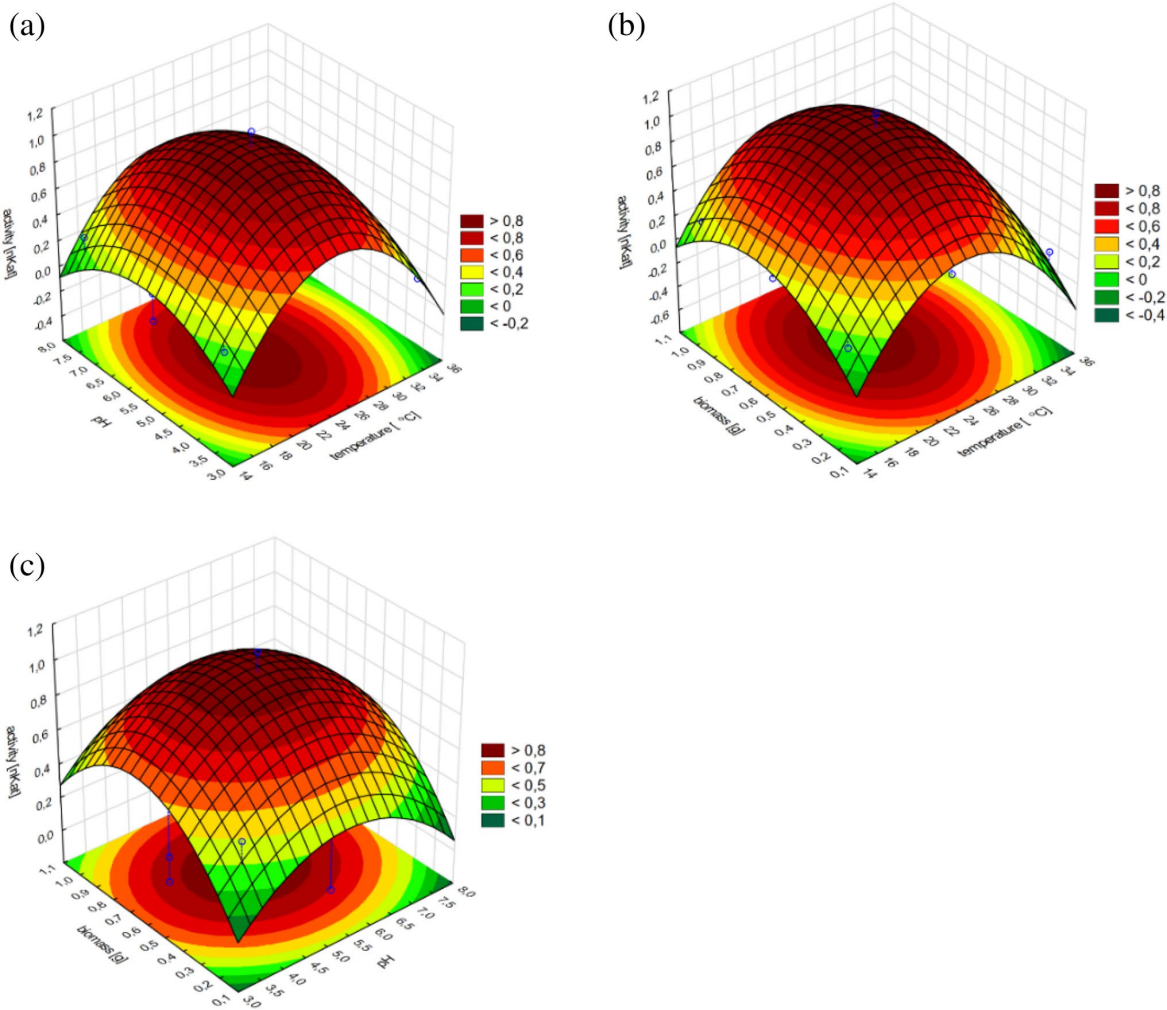
Response surface plots fitted to the model describing chitinase production by *T. citrinoviride* B3, with regard to combined effects of (**a**) culture medium pH and cultivation temperature; **b** addition of fungal biomass and cultivation temperature; **c** addition of fungal biomass and pH of the medium

**Fig. 6:**
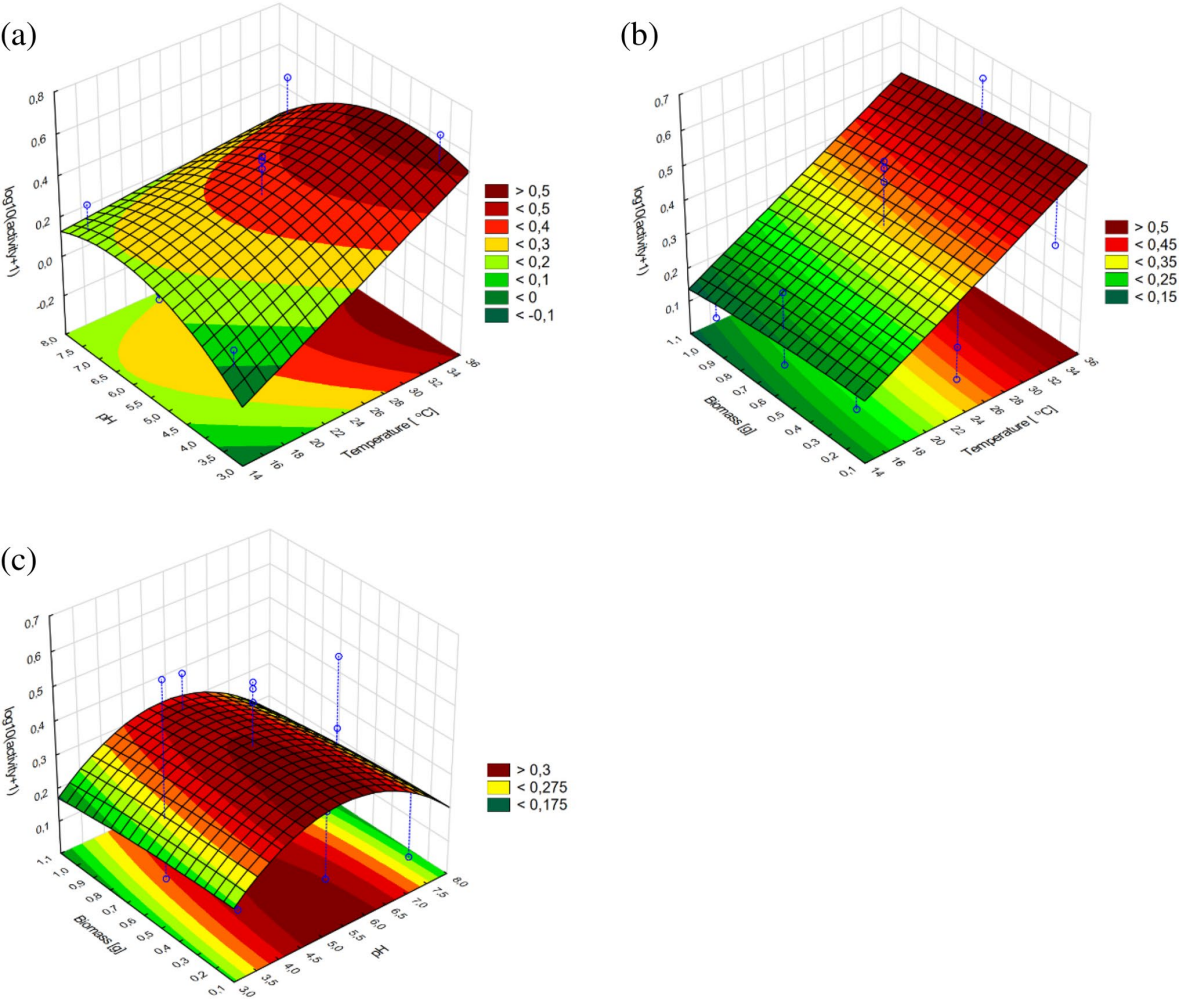
Response surface plots fitted to the model describing chitinase production by *T. citrinoviride* C1 with regard to (**a**) culture medium pH and cultivation temperature; **b** addition of fungal biomass and cultivation temperature; **c** addition of fungal biomass and pH of the medium

The activity of chitinase was dependent on each of the tested variables, but the impact of culture temperature was strictly linear, within the experimental range. The correlation of biomass addition and the response was characterized by an increasing trend; therefore, the current optimum was established as the layout boundary. Nevertheless, the optimal pH value could be precisely determined (Figs. 5, 6).

A high suitability of the regression models was obtained, as indicated by coefficients of determination *R*^2^ = 0.9582 and *R*^2^ = 0.9163, in the case glucanases of the B3 and C1 strain, respectively, as well as R^2^ = 0.9477 and R^2^ = 0.9594 in the case of chitinases (Tables 6, 7). This implied that the models explained at least 91% of response variability, while the remaining percentage of variability was attributed to noise. The Fisher’s test *F*-values that verified the high significance of the models fell in the range between *F* = 41.79 for chitinases of the C1 strain and *F* = 10.65 for glucanases of the same strain. Each of the models passed the “lack of fit” test of insignificant rank that further confirmed its correctness.

**Table 6:** Analysis of variance (ANOVA) of regression models for glucanase production

Error source	Sum of squares (SS)	Degrees of freedom (DF)	Mean square (MS)	*F*-value	*p*-value
Strain B3: *R*^2^ = 0.9582, *R*^2^_adj_ = 0.9163					
Regression Model	2.3071	7	0.3296	22.89	< 0.001
Residual error	0.1008	7	0.0144	-	-
Lack of fit	0.0967	5	0.0193	9.44	0.0986
Pure error	0.0041	2	0.0020	-	-
Cor. total	2.4079	14	-	-	-
Strain C1: *R*^2^ = 0.9163, *R*^2^_adj_ = 0.8842					
Regression Model	2.8103	7	0.4015	10.65	< 0.01
Residual error	0.2261	6	0.0377	-	-
Lack of fit	0.2224	7	0.0318	17.14	0.0562
Pure error	0.0037	2	0.0019	-	-
Cor. total	3.0364	14	-	-	-

**Table 7:** Analysis of variance (ANOVA) of regression models for chitinase production

Error source	Sum of squares (SS)	Degrees of freedom (DF)	Mean square (MS)	*F*-value	*p*-value
Strain B3: *R*^2^ = 0.9477, *R*^2^ adj = 0.9084					
Regression Model	1.9468	6	0.3245	24.04	< 0.001
Residual error	0.1076	8	0.0135	-	-
Lack of fit	0.0875	6	0.0146	1.45	0.4618
Pure error	0.0201	2	0.0100	-	-
Cor. total	2.0544	14	-	-	-
Strain C1: *R*^2^ = 0.9594, *R*^2^_adj_ = 0.9369					
Regression Model	0.5852	5	0.1170	41.79	< 0.001
Residual error	0.0248	9	0.0028	-	-
Lack of fit	0.0230	7	0.0033	3.7128	0.2285
Pure error	0.0018	2	0.0009	-	-
Cor. total	0.6100	14	-	-	-

The conducted experiments allowed us to observe a significant discrepancy between the tested strains belonging to the same species. The higher temperature had the greatest effect on the biosynthesis of chitinases by the C1 strain in combination with 0.5 g of fungal biomass in the substrate and the pH of the substrate of 3.0. The least optimal conditions for culture for chitinase biosynthesis by *Trichoderma citrinoviride* C1 turned out to be neutral pH at a culture temperature of 15 °C with an insufficient or excessive amount of biomass in the substrate. The variable pH value had no effect on chitinase biosynthesis in culture at 15 °C with low fungal biomass content in the medium. The highest biosynthesis of glucanases by the C1 strain was influenced by the high biomass content in the substrate at pH 8.0. The biosynthesis of glucanases of this strain increased along with increasing biomass content in the culture medium. It reached its highest value at ~ 28 °C. At temperature of 26–28 °C, the most effective biosynthesis of this enzyme occurred in the broad pH range. Temperature below 20 °C adversely affects the biosynthesis of glucanases by the C1 strain. Increasing the addition of biomass in substrate conditions at pH 7.5 resulted in a gradual increase in chitinase biosynthesis by the B3 strain. With the increased addition of fungal biomass in the substrate and the decrease in culture temperature, the biosynthesis of chitinases by the B3 strain increased. The dependence of culture temperature on the pH of the substrate in the biosynthesis of this enzyme is very small. In order to obtain the best possible biosynthesis of β-1–3 and β 1–4-glucanase, it seems important to increase the biomass content in the substrate from pH ~ 6.0, along with increasing the culture temperature to 36 °C. Temperature below 20 °C, regardless of the amount of fungal biomass added to the substrate, adversely affects the biosynthesis of glucanases by the B3 strain (Tables 8, 9).

**Table 8:** Summary of effects from reduced models for glucanase

Variable	Coefficient	Standard error	*t*-value	*p*-value
*T. citrinoviride* B3				
Intercept	0.7071	0.0131	54.1193	0.0003
X_1_	0.6025	0.0320	18.8242	0.0028
X_2_	0.3889	0.0320	12.1509	0.0067
X_3_	0.1715	0.0320	5.3598	0.0331
X_1_X_1_	0.4006	0.0236	17.0089	0.0034
X_2_X_2_	0.3604	0.0236	15.3010	0.0042
X_3_X_3_	− 0.1017	0.0236	− 4.3167	0.0497
X_1_X_2_	0.3645	0.0453	8.0531	0.0151
*T. citrinoviride* C1				
Intercept	0.7050	0.0119	59.0435	0.0003
X_1_	0.6839	0.0304	22.4667	0.0020
X_3_	0.4878	0.0304	16.0248	0.0039
X_1_X_1_	0.5930	0.0223	26.5462	0.0014
X_3_X_3_	0.1463	0.0223	6.5476	0.0225
X_1_X_2_	− 0.2286	0.0431	− 5.3106	0.0337

**Table 9:** Summary of effects from reduced models for chitinase

Variable	Coefficient	Standard error	*t*-value	*p*-value
*T. citrinoviride* B3				
Intercept	0.2064	0.0289	7.1386	0.0191
X_1_	− 0.1590	0.0708	− 2.2449	0.1539
X_2_	− 0.0245	0.0708	− 0.3459	0.7624
X_3_	0.1440	0.0708	2.0331	0.1791
X_1_X_1_	0.6440	0.0521	12.3533	0.0065
X_2_X_2_	0.2340	0.0521	4.5265	0.0455
X_3_X_3_	0.2905	0.0521	5.5720	0.0307
*T. citrinoviride* C1				
Intercept	0.2442	0.0082	29.6038	0.0011
X_1_	0.3385	0.0210	16.0972	0.0038
X_2_	− 0.0125	0.0210	− 0.5929	0.6133
X_2_X_2_	0.1216	0.0154	7.8800	0.0157
X_3_X_3_	0.2766	0.0154	17.9261	0.0031
X_1_X_2_	− 0.1810	0.0297	− 6.0861	0.0260

The obtained results allowed to define polynomial equations to illustrate reduced regression models. Equation 2 was fitted to the model describing the production of glucanase by *T. citrinoviride* B3, and Eq. 3 was matched to that of *T. citrinoviride* C1.

2 $$Y= -4.02 + 0.18{X}_{1}+ 0.86{X}_{2}- 0.55{X}_{3}- 0.00{X}_{1}{X}_{1}- 0.09{X}_{2}{X}_{2}+ 0.64{X}_{3}{X}_{3}+ 0.01{X}_{1}{X}_{2}$$

3 $$Y = -4.06 + 0.33{X}_{1} + 1.70{X}_{3}- 0.01{X}_{1}{X}_{1}- 0.91{X}_{3}{X}_{3}- 0.00{X}_{1}{X}_{2}$$

For the production of chitinase, the following equations were fitted for the models with B3 and C1 strain (Eqs. 3 and 4), respectively:

4 $$Y = -5.35 + 0.31{X}_{1}+ 0.64{X}_{2}+ 2.36{X}_{3}- 0.01{X}_{1}{X}_{1}- 0.06{X}_{2}{X}_{2}- 1.82{X}_{3}{X}_{3}$$

5 $$Y = -1.43 + 0.04{X}_{1}+ 0.39{X}_{2}- 0.03{X}_{2}{X}_{2}- 0.041{X}_{3}{X}_{3}- 0.00{X}_{1}{X}_{2}$$

## Discussion

For the development of microorganisms, not only certain nutrients are necessary but also appropriate environmental conditions, such as appropriate concentration of hydrogen ions, temperature, and adequate exposure to oxygen (Mohiddin et al 2021). These parameters typically undergo optimization since they considerably affect microbial growth and biosynthesis of secondary metabolites (Bednarski et al. 2003). The research carried out in the presented work, employing *Trichoderma citrinoviride* filamentous fungi, involved modelling the biosynthesis level of 1,3 and β-1,4 glucanases and chitinases as a function of selected cultivation conditions, i.e., supplementation with fungal biomass as an inducer of enzymes and an important source of carbon in the substrate, its pH and cultivation temperature. This allowed for determining the optimal levels for these parameters to maximize the desired production of hydrolyses. This seems particularly important, given the continuing interest in the issue of fungal chitinases (Thakur et al. 2023).

In a previous study (Piegza et al. 2014), it has been demonstrated that either the addition of purified cell walls of microorganisms or biomass containing chitin/laminarin induced biosynthesis of hydrolytic enzymes by *Trichoderma* sp. It was demonstrated that the cell wall biomass of filamentous fungi and yeast contains 23–28% β-glucan and 8–11% chitin in d.m. Insect biomass was considered an alternate source of chitin, in which up to 45% of this polymer is found. Hence, insects can serve as an excellent source of carbon and energy, as well as an inducer of lytic enzyme biosynthesis in *Trichoderma* cultures (Piegza et al. 2014). In this study *T. harzianum* T33 and *T. citrinoviride* C1 cultivated on media with the addition of biomass from various filamentous fungi, including plant pathogens (*Alternaria, Aspergillus, Mucor, Rhizopus, Absidia,* and *Botrytis*) and yeast (*Geotrichum*), an increase in the biosynthesis of chitinases of strains was observed. Supplementation of culture medium with insect biomass (*Musca*) resulted in more efficient expression of laminarinase in the C1 strain.

The biochemical characterization of a commercial mixture of chitinolytic enzymes derived from *T. viride* was carried out, and their effect in vitro and in vivo on the peritrophic matrix (PM) of the silkworm *Bombyx mori* was demonstrated by Berini et al. (2016). *Trichoderma citrinoviride* strains with significant chitinolytic activity were used in composting of i.a. chitosan derivatives, also proved its importance from the point of view of not only biocontrol, but sustainable agriculture in general (Swiontek-Brzezińska et al. 2023).

These enzymes were found to exert a significant in vitro effect on the PM structure and permeability. Although the T. *viride* chitinase mixture was more active in the degradation of chitin at acidic pH, it was shown that the residual activity retained at higher pH was sufficient for its action in vivo, within the PM. Berini et al. (2016) established that the tested enzymes could find prospective application in pest control, that is, they can be used to target the chitinous structures of insects and to degrade cell walls of filamentous fungi. The authors denoted that other fungal chitinases could potentially exhibit higher activity. It has been demonstrated that the majority of known fungal chitinases of different species are effectively produced at similar pH, i.e., in the range of pH 4.0–7.0, at temperatures 20–40 °C. Nevertheless, some of the enzymes, despite their acidic or slightly acidic pH optima, also retain remarkable hydrolytic activity in a moderately alkaline environment (Berini et al. 2016). It is worth noting that other *Trichoderma* species also exhibit high chitinase biosynthesis in a near-neutral environment and at a temperature of 30 °C. Such conditions, along with the significant impact of the carbon and nitrogen source, were indicated by Gueye et al. (2020).

Kancelista et al. (2015) in the research involving the *T. citrinoviride* C1 strain reported enzymatic activity of approximately 0.5 nKat/mL, after 3 days of agitated culture conducted at 28 °C and pH 5.0. This activity was seven times lower than that obtained in the described studies. Taking into account the biosynthesis of glucanases of the C1 strain, the verified optimal culture conditions were: cultivation temperature of 28 °C, fungal biomass content of 0.9 g, and broad range of pH. The activity under these conditions on the third day of cultivation was 26.67 nKat/mL. However, according to Kancelista et al. (2015), a result of approximately 11.67 nKat/mL was obtained, but a similar value was demonstrated on the fifth day of cultivation ~ 29.17 nKat/mL.

## Conclusion

Based on the presented results, it can be concluded that the *T. citrinoviride* C1 strain is a superior producer of glucanases and chitinases, that may be applicable in the form of inoculations to protect plants against pathogens. Under the following culture conditions: temperature 35 °C, pH 3.5, and 0.6 g/L fungal biomass in the medium, *Trichoderma citrinoviride* C1 biosynthesized chitinases at the 3.55 nKat/mL level. Biosynthesis β-1.3 and β-1.4 glucanases reached the highest level for the *T. citrinoviride* C1 strain at culture temperature 25 °C, pH 7.5, and the addition of 1.0 g/L of fungal biomass.

## Data Availability

The data and materials that support the findings of this study are available from the corresponding author on request.
